# Resident and transient coyotes exhibit differential patterns of movement behavior across heterogeneous landscapes in the southeastern United States

**DOI:** 10.1002/ece3.8725

**Published:** 2022-03-22

**Authors:** Sarah C. Webster, James C. Beasley, Joseph W. Hinton, Michael J. Chamberlain

**Affiliations:** ^1^ 1355 Savannah River Ecology Laboratory Warnell School of Forestry and Natural Resources University of Georgia Aiken South Carolina USA; ^2^ Wolf Conservation Center South Salem New York USA; ^3^ 1355 Warnell School of Forestry and Natural Resources University of Georgia Athens Georgia USA

**Keywords:** *Canis latrans*, first passage time, space use, transiency

## Abstract

Coyotes (*Canis latrans*) are a highly adaptable canid species whose behavioral plasticity has allowed them to persist in a wide array of habitats throughout North America. As generalists, coyotes can alter movement patterns and change territorial strategies between residency (high site fidelity) and transiency (low site fidelity) to maximize fitness. Uncertainty remains about resident and transient coyote movement patterns and habitat use because research has reached conflicting conclusions regarding patterns of habitat use by both groups. We quantified effects of habitat on resident and transient coyote movement behavior using first passage time (FPT) analysis, which assesses recursive movement along an individual's movement path to delineate where they exhibit area‐restricted search (ARS) behaviors relative to habitat attributes. We quantified monthly movement rates for 171 coyotes (76 residents and 53 transients) and then used estimated FPT values in generalized linear mixed models to quantify monthly habitat use for resident and transient coyotes. Transients had greater movement rates than residents across all months except January. Resident FPT values were positively correlated with agricultural land cover during fall and winter, but negatively correlated with agriculture during spring. Resident FPT values were also negatively correlated with developed habitats during May–August, deciduous land cover during June–August, and wetlands during September–January except November. FPT values of transient coyotes were positively correlated with developed areas throughout much of the year and near wetlands during July–September. Transient FPT values were negatively correlated with agriculture during all months except June and July. High FPT values (ARS behavior) of residents and transients were generally correlated with greater densities of edge habitat. Although we observed high individual variation in space use, our study found substantive differences in habitat use between residents and transients, providing further evidence that complexity and plasticity of coyote habitat use is influenced by territorial strategy.

## Graphical abstract

We utilized first passage time analysis to assess differences in movement patterns of resident and transient coyotes in the southeastern United States. Although we observed high individual variation in space use and habitat selection, our study found substantive differences in habitat selection between resident and transient coyotes, providing further evidence that complexity and plasticity of coyote habitat selection is influenced by social status.

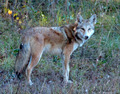

## 1 INTRODUCTION

Coyotes (*Canis latrans*) are a highly adaptable canid whose behavioral plasticity has allowed them to persist in a wide array of habitats and climates, ranging from relatively undisturbed natural areas to highly developed urban environments (DeCandia et al., 2019; Gerht et al., 2009; Gompper, 2002). As opportunistic generalists, coyotes are able to switch among various food resources (Patterson et al., 1998; Randa et al., 2009), adjust their movement patterns to minimize conflicts with conspecifics, other predators, and humans (Berger & Gese, 2007; Fedriani et al., 2001), and change individual social strategies to maximize survival and reproduction (Macdonald, 1983). These characteristics have facilitated an extensive range expansion and growth of coyote populations over the past century, while other canid populations have declined (Hinton et al., 2019).

Range expansion of coyotes has had several impacts on newly colonized ecosystems, including altering prey population dynamics (Crimmins et al., 2012; Kilgo et al., 2010; Waser et al., 2014) and increasing interference competition for resources among established predator populations (Berger & Gese, 2007; Harrison et al., 1989; Johnson et al., 1996). Many of these observed trends are thought to be density dependent, with impacts becoming more pronounced as coyote populations increase and animals saturate the landscape (Gompper, 2002). As a result, managers and researchers recognize the need for a more comprehensive understanding of coyote spatial ecology, particularly territoriality and habitat selection, in recently colonized regions.

Adult coyotes typically exhibit one of two patterns of territorial space use: residency or transiency. Residents maintain small, mutually exclusive home ranges as breeding pairs, whereas transients typically move across landscapes without a social group and often overlap with other individuals’ home ranges (Gese, 2004; Hinton et al., 2015; Kamler & Gipson, 2000; Morin & Kelly, 2017). Territorial status has substantive implications for how coyotes interact with their surrounding environments, including habitat use and prey selection (Mills & Knowlton, 1991; Ward et al., 2018). Transient coyotes differ from residents because they are individuals who typically move alone, exhibit low site fidelity, and do not breed (Carmenzind, 1978; Hinton et al., 2015; Kamler & Gipson, 2000). Because they maintain territories with mates, residents have greater foraging success (Gese et al., 1996) and lower mortality rates (Knowlton et al., 1999) than do transients. Recent research on the spatial ecology of transient coyotes has focused on the space use (i.e., biding areas; Hinton et al., 2012, 2015) and behaviors (i.e., biding; Morin & Kelly, 2017) prior to transients establishing residency.

Several studies have investigated coyote space use and habitat selection, but relatively few have differentiated selection between resident and transient behaviors when conducting their analyses. Of those that made this differentiation, all noted that resident coyotes were found to select for open grassland, pasture, and agricultural habitats while avoiding developed habitats (Hinton et al., 2015; Kamler & Gipson, 2000). However, patterns of habitat selection for transient coyotes are more ambiguous. Kamler and Gipson (2000) found transients avoided grasslands and selected woodlands, whereas Hinton et al. (2015) found transient coyotes exhibited similar selection trends to residents by selecting open habitats, although transients were more likely to use roads than residents. Transient coyotes have also been documented using habitats associated with human development (Gerhrt et al., 2009; Mitchell et al., 2015). Notably, previous studies faced logistical and practical limitations in sample sizes or data resolution (VHF vs. GPS technology) that may have impacted observed trends (Hinton et al., 2015). Additionally, most previous research has quantified habitat selection by both residents and transients based on an individual's estimated home range (e.g., 3rd‐order resource selection functions [RSF]), an approach that may not be appropriate for transient coyotes who do not have stable home ranges over time (Morin & Kelly, 2017). For species who do not maintain stable home ranges, characterization of movement behaviors along an individual's movement path and association of those behaviors with the habitats in which they occur may be a more appropriate approach to determine habitat selection.

One such approach, first passage time (FPT) analyses (Fauchald & Tverra, 2003), allows for fine‐scale delineation of where an animal is spending time by estimating when an individual is exhibiting area‐restricted search behavior (ARS; i.e., slow travel speed and high tortuosity) along its movement path. Low FPT values are associated with faster linear movements (non‐ARS behavior), whereas higher FPT values indicate an animal's movements are slower and more sinuous (ARS behaviors). By using FPT analyses, researchers can assess residency time based on where an animal is engaging in ARS behaviors (e.g., foraging) vs. non‐ARS behavior (e.g., traveling), and these methodologies have successfully been used previously to investigate fine‐scale habitat selection of other mesocarnivores such as raccoons (*Procyon lotor*; Fauchald & Tverra, 2003; Byrne & Chamberlain, 2012). Additionally, FPT analyses do not rely on estimated home ranges required by traditional resource selection methodologies, ultimately reducing uncertainty in inferred patterns of correlation between ARS behaviors and environmental characteristics, especially for individuals that do not maintain home ranges.

Thus, our goal was to quantify the relationships between habitat characteristics and ARS behaviors of resident and transient coyotes across the southeastern United States using FPT analyses to distinguish spatiotemporal patterns of residency time for both groups. Because resident and transient coyotes are known to exhibit different preferences for land cover types (Hinton et al., 2015; Kamler & Gipson, 2000), we hypothesized that the differing territorial strategies and movement behaviors of resident and transient coyotes would influence space use and FPT values in relation to various land cover types. We predicted that transient coyote ARS behaviors (i.e., high FPT values) would be positively correlated with land cover types associated with travel corridors, such as human development and edge habitats, both of which have been found important to transient coyotes in previous studies (Gerhrt et al., 2009; Hinton et al., 2015). Contrarily, we expected ARS behaviors (i.e., high FPT values) of resident coyotes to be negatively correlated with human development but correlated with open land cover (e.g., agriculture) that were reported to be important land cover preferred by coyotes (Hinton et al., 2015; Kamler & Gipson, 2000). Finally, because resident coyotes form breeding pairs to defend territories and raise offspring whereas transient coyotes are solitary animals primarily dispersing from natal areas, we hypothesized that differences in resident and transient reproductive behaviors would affect spatiotemporal patterns in movement rates. We predicted that resident coyotes would exhibit reduced movement rates relative to transients during months when they were likely to be raising offspring.

## 2 METHODS

### 2.1 Study area

**FIGURE 1:**
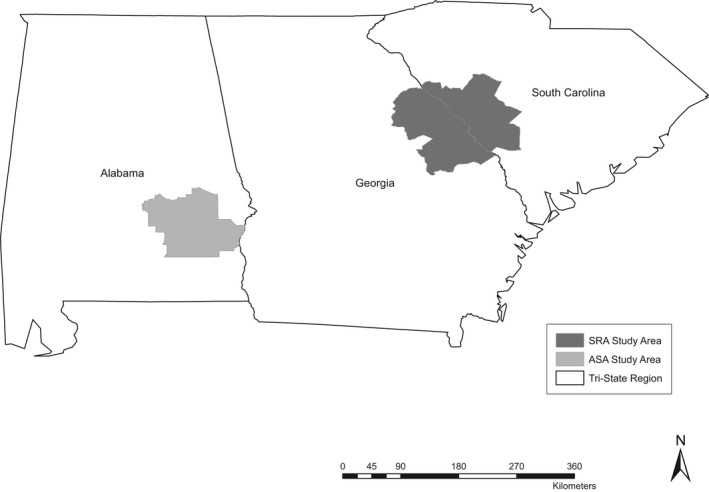
Alabama study area (ASA) and the Savannah River study area (SRA), located in Alabama, Georgia, and South Carolina, USA, where coyotes were captured and monitored with GPS collars during 2015–2017

Our study area included regions of Alabama (Barbour, Macon, and Pike Counties), Georgia (Columbia, Jefferson, Lincoln, McDuffie, and Warren Counties), and South Carolina (Aiken, Barnwell, Edgefield, McCormick, and Saluda Counties) in the southeastern United States, totaling approximately 16,200 km^2^ (Figure 1). Coyotes captured in Georgia and South Carolina commonly moved between the respective study areas and likely represented one population, leaving two distinct study areas: the Alabama study area (ASA) and the Savannah River study area (SRA) of Georgia and South Carolina. Both study areas were comprised predominantly of privately owned land, but approximately 20% of the SRA was comprised of the Savannah River Site (SRS), an 803 km^2^ federal facility operated by the U.S. Department of Energy (DOE). Both study areas had mild subtropical climate throughout the year. Summers were generally hot and humid with an average high temperature of approximately 30°C, whereas winters were mild with an average low temperature of approximately 1°C (National Oceanic and Atmospheric Administration (NOAA), 2019). Habitats in both the ASA and the SRA were a mix of successional forest, agriculture, pastureland, pine plantations, and urban habitats. Agriculture in these regions included cotton (*Gossypium* spp.), corn (*Zea mays*), tobacco (*Nicotiana tabacum*), soybeans (*Glycine max*), and peanuts (*Arachis hypogaea*). For further details on our study areas see Ward et al. (2018).

### 2.2 Data collection

We deployed GPS collars on coyotes over three fall/winter seasons in 2015, 2016, and 2017. We captured animals with foothold traps (Victor #3 Softcatch, Woodstream Corporation, Lititz, Pennsylvania, USA; MB 550 or MB 450, Minnesota Trapline Products, Pennock, Minnesota, USA) with offset or padded jaws. During 2015–2016, animals were restrained with a catchpole, muzzle, and hobbles for processing. During 2017, we used chemical immobilization in addition to physical restraint because we collected biological samples (e.g., blood, feces, and parasites) in addition to fitting each animal with a collar. By using chemical immobilization when collecting these additional samples, we were able to minimize stress to the animal and reduce processing time. We anesthetized animals prior to processing using a ketamine/xylazine mixture administered at 0.8 ml/kg for ketamine and 0.1 ml/kg for xylazine. We then determined sex, weight, and age using tooth wear (Gipson et al., 2000). Coyotes >2 years old were considered adults, whereas 1–2‐year olds were considered juveniles, and animals <1 year old were classified as pups. We fitted each animal with a mortality‐sensitive satellite collar (either G2110E Iridium collar, Advanced Telemetry Systems, Isanti, Minnesota, USA or Litetrack Iridium collar, Lotek Wireless Inc., New Market, Ontario, Canada). Collars recorded locations at a 4‐h interval. Prior to release, we administered anesthetized animals yohimbine at 1.0 ml/kg. All animal handling procedures were approved by the University of Georgia Institutional Animal Care and Use Committee (protocols A2014 08‐025‐R2 and A2015 05‐004‐A5). To access lands to trap, state agencies and the DOE granted permission for publicly owned property while we obtained permission from landowners to access privately owned lands.

### 2.3 Movement data analysis

To determine territorial status of collared animals, we used a combination of ≥3 months of space use by coyotes (Hinton et al., 2015) and a rarefaction curve for each animal created by calculating monthly home ranges (Dellinger et al., 2013). Previous studies have found that resident coyotes in the southeastern U.S. maintain home ranges that range from approximately 5 to 45 km^2^ (Hinton et al., 2015, Mastro et al. 2019). Thus, we classified resident coyotes as animals that showed stable space use for ≥3 months and had home ranges smaller than 45 km^2^. Following Hinton et al. (2015), we classified transients as animals with ranges larger than 45 km^2^ who exhibited unstable space use over time. We estimated 95% home ranges and transient ranges and 50% core areas and biding areas using fixed kernel density with the reference (href) smoothing parameter (Worton, 1989). Using both methods for identifying territorial status allowed for confident classification of residents and transients, but also meant that we were unable to determine territorial status of animals with <3 months of movement data due to mortality or collar failure. If we were unable to determine territorial status for an individual, it was excluded from further analysis. For transient animals, we refer to space use patterns as biding areas because transients do not maintain territories (Hinton et al. 2012, 2015; Morin & Kelly, 2017).

Previous research has shown that coyote space use varies seasonally due to a variety of biological and ecological attributes (Hinton et al., 2015; Kamler & Gipson, 2000; Sasmal et al., 2019). However, the criteria researchers use to define ecologically or biologically relevant seasons typically varies among studies (e.g., seasons defined by environmental conditions vs. organism behaviors) depending upon the research question, data resolution, and study duration. Variation in season delineation can potentially bias results or mask important trends in spatial data (Basille et al., 2013; Thompson & McGarigal, 2002). To mitigate this issue, we decided to conduct all spatial analyses by month. Quantifying movement on a monthly basis allowed us to minimize potential bias due to misclassification of relevant seasons. We also quantified average movement rates for both resident and transient coyotes by dividing step length between two consecutive locations by the time interval (4 h) between those locations and compared movement rates per hour across months. Only locations with approximate 4‐hour time intervals (with a ~3‐min buffer allowed to account for occasional lags in satellite data transfer times) were included in analysis to minimize error associated with missing data. To determine whether movement behaviors between the two classes differed temporally, we used generalized linear mixed models (GLMMs) where movement rate was the response variable and territorial status (resident or transient) was the predictor variable. We included individual coyote as a random effect in all models and modeled each month separately. An alpha value of 0.05 was used to determine significance in all statistical tests.

We used FPT analyses following Fauchald and Tverra (2003) to quantify relationships between landscape features and monthly coyote space use and movement behaviors. FPT is the time required for an animal to cross a circle of a given radius (Johnson et al., 1992) and can be used to infer movement behaviors and inform residency times when FPT values are estimated along an individual's movement path. Low FPT values are associated with faster linear movements, whereas higher FPT values indicate an animal's movements are slower and more sinuous. More sinuous movements are inferred as ARS behavior, often associated with foraging or loafing behaviors. Thus, researchers are able to differentiate between different behavioral states (i.e., traveling vs. foraging/loafing) and quantify which habitats these behaviors occur within. We analyzed movement paths from resident and transient coyotes on a monthly basis by subsetting movement data by month and requiring an individual to have a minimum of 90 relocations within a month to be included in each monthly analysis. To determine the appropriate scale at which to estimate FPT values, we first interpolated locations every 20 m along movement paths and calculated FPT values at these locations for circles with radii ranging from 10 to 4000 m in 10 m increments. We then calculated the variance of log‐transformed FPT values for each trajectory and circle radius to determine at which radius the variance peaked, indicating the scale at which individuals were concentrating ARS behaviors (Fauchald & Tverra, 2003). This scale varied across individual movement paths, so we calculated an average scale across all individuals for each month for comparisons (Byrne & Chamberlain, 2012; Freitas et al., 2008). We then recalculated FPT values for all individuals using the averaged radius size for each month. By estimating FPT values at differing scales monthly and only including individuals which met robust data thresholds, we minimized bias introduced by seasonal and individual variation in movement patterns.

### 2.4 Habitat analyses

We assessed habitat composition of the study areas using a 30‐m resolution National Land Cover Database (NLCD) 2011 land cover raster layer. Using Spatial Analyst in ArcMap 10.3, we reclassified the NLCD raster layer into six primary land cover types: mixed deciduous forest, pine forest, wetland, agriculture, and developed. Because coyotes are known to use edge habitats (i.e., the boundary between two land cover types; Heske et al., 1999; Hinton et al., 2015; Tigas et al., 2002), we also calculated edge density within each habitat class using package “landscapemetrics” in Program R (Hesselbarth et al., 2019; R Core Team, 2018).

To determine which land cover characteristics were associated with ARS behaviors, we measured the distance of each location along an individual's movement path to each land cover type and quantified average edge density within a 100 m radius around each location. A distance‐based approach combined with a consistent measure of edge density at each location allowed for consistent quantification of an individual's spatial relationship to habitat covariates of interest (Benson, 2013) even as the scale at which FPT values were estimated varied across months. We then used a GLMM to determine whether areas with high FPT values (i.e., areas where individuals were engaging in ARS behaviors) were associated with particular land cover characteristics. We included FPT values as a continuous response variable in all models. Often, FPT values are reduced into two binary, categorical variables of high (ARS) and low (non‐ARS) values (Fauchald & Tverra, 2003). However, given the high level of individual variation we observed in sampled individuals, particularly among transient coyotes, creating a discrete threshold between FPT values in order to create a binary variable would likely introduce bias into our model interpretations. By quantifying FPT values as a continuous variable, we mitigated this potential bias and ultimately allowed for more nuanced interpretation of model outputs. We modeled resident and transient animals separately for each month, so the scale of FPT estimated values was consistent for all data included in a model. For both classes of coyote in each month, we ran a suite of six GLMMs with all land cover variables (mixed deciduous forest, pine forest, wetland, agriculture, developed, and edge habitat) and all biologically relevant subsets to test our predictions of resident and transient FPT values associated with various habitat types (Appendix 1). By including models with potentially biologically relevant variable subsets, we allowed for thorough analysis of the impacts of all six primary land cover types on coyote movement behaviors beyond those specifically identified in our predictions. Given the broad variation we observed among individuals, this conservative approach allowed us to be confident that the top‐ranked models were not only top ranked because a biologically important variable combination was excluded from analysis. In all models, we included individual coyote as an additive random effect to account for spatial and temporal autocorrelation between each individual's movement data. To avoid multicollinearity, we examined correlations among model variables by deriving a matrix of all possible Spearman correlation coefficient values. Any variables with a significant correlation (*r* ^2^ > .6; *p* < .05) were not simultaneously included in the same model in subsequent analysis. We also used variance inflation factor (VIF) to confirm variables were not displaying collinearity or instability (VIF > 5; Dormann et al., 2013; Kutner et al., 2004) and found no evidence of collinearity as all VIF was less than 2. We associated ARS behaviors with a particular land cover type when locations with high FPT values were significantly closer in distance (meters) to certain land cover types than locations with low FPT values. We then used Akaike's information criterion (AIC) to compare models and used the most parsimonious model to estimate model parameters, including beta coefficients (β), of correlation of habitat characteristics to ARS behaviors within the model. In the event that >1 model was within 2 AIC units of the top model, we model averaged to derive parameter estimates (Burnham & Anderson, 2002). We conducted all statistical analyses in Program R (R Core Team, 2018).

## 3 RESULTS

**FIGURE 2:**
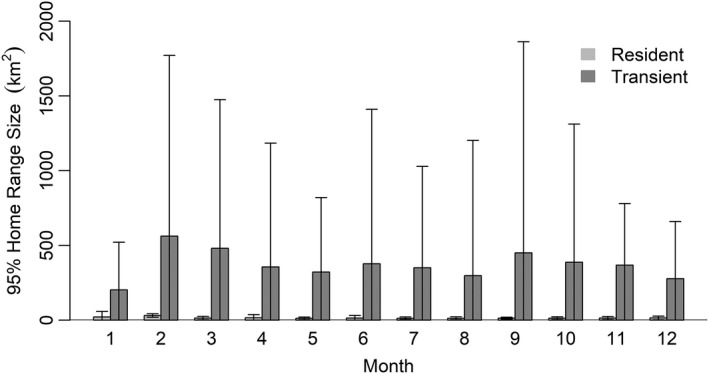
Mean monthly 95% home range or biding area estimates for resident and transient coyotes, respectively

**FIGURE 3:**
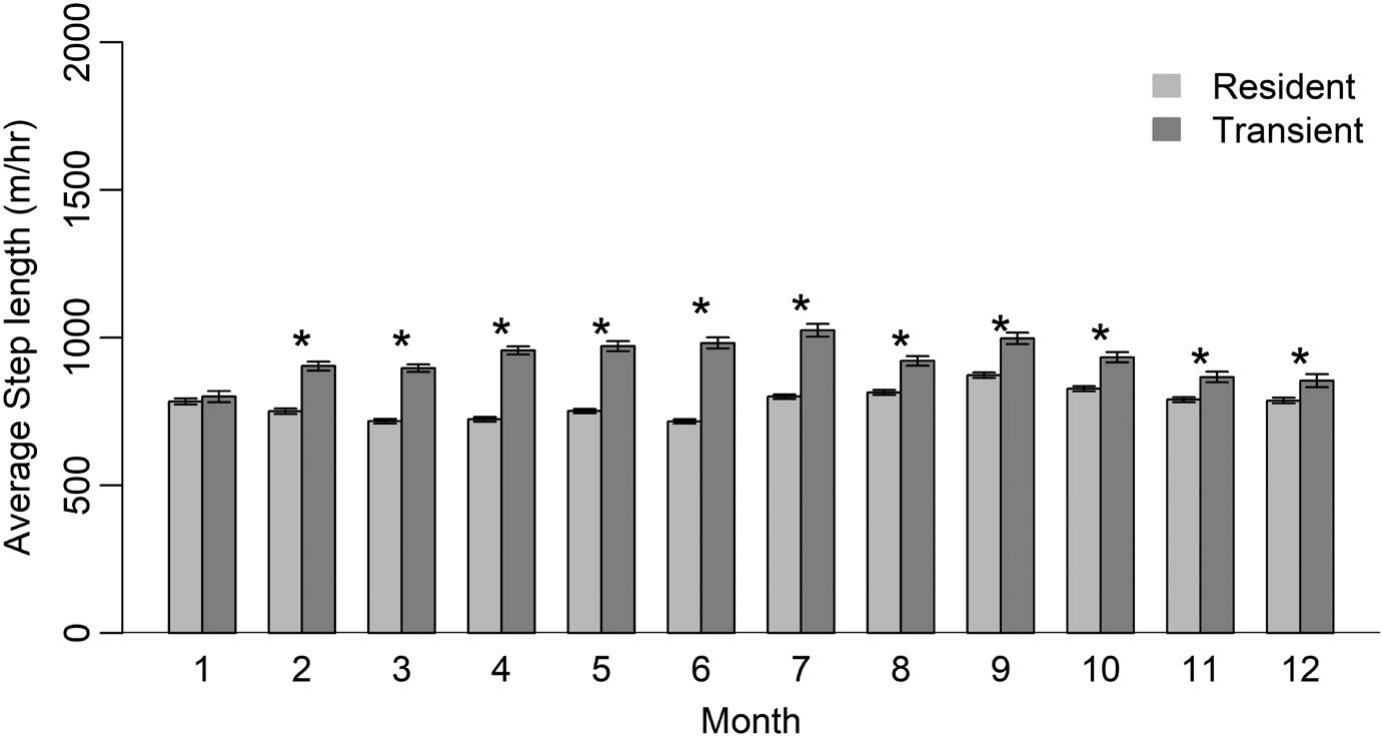
Average monthly movement rate for resident and transient coyotes monitored from January 2015 to June 2017 in the tristate region of Alabama, Georgia, and South Carolina. Error bars shown represent standard error, and asterisks denote significant differences between groups

We deployed collars on 193 coyotes, 54 in the ASA and 139 in the SRA. We excluded 22 coyotes from analysis due to an insufficient number of relocations. Of the remaining 171 coyotes, 76 (44.4%) were residents and 53 (30.1%) were transients for the entire time they were monitored, whereas 42 (24.6%) exhibited both residency and transiency. We included individuals who were both residents and transients at different time periods during monitoring in analyses, but separated their movement paths into different paths during residency and transiency. Mean monthly 95% home range size for residents was 15.16 km^2^ (*SD* = 21.88 km^2^) and ranged from 11.18 to 30.51 km^2^, while mean 95% transient range size for transients was 368.81 km^2^ (*SD* = 799.80 km^2^) and ranged from 202.14 to 561.44 km^2^ (Figure 2; Appendix 2). Movement rates varied between residents and transients across all months except January and December, with transients generally having greater movement rates than residents (Figure 3; Appendix 3).

**FIGURE 4:**
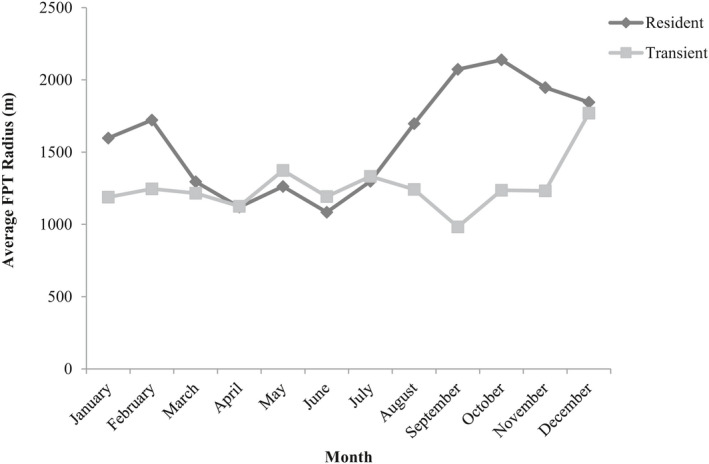
Average estimated radius at which first passage time (FPT) values were calculated each month for resident and transient coyotes in the tristate region of Alabama, Georgia, and South Carolina

**FIGURE 5:**
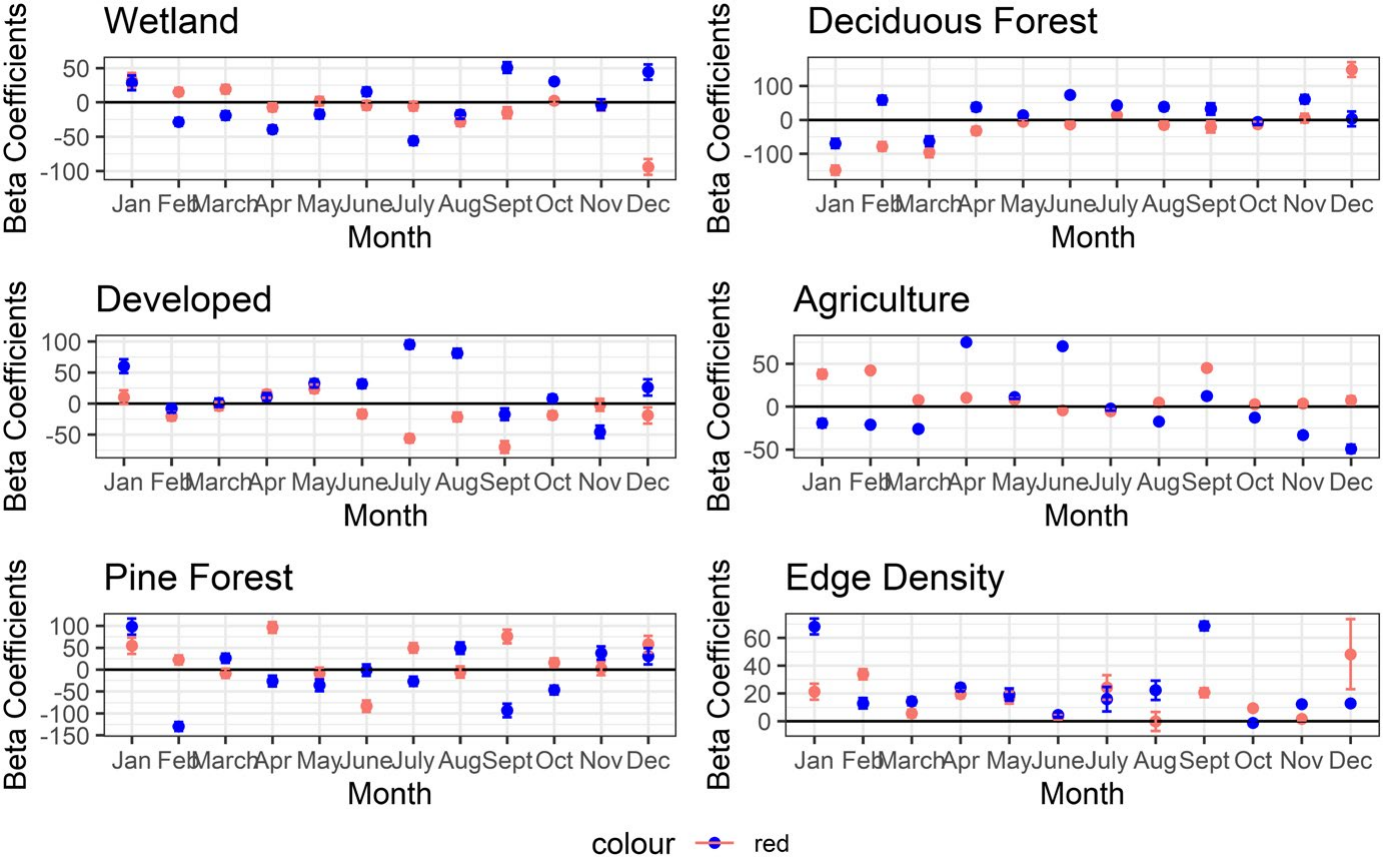
Beta coefficient estimates of habitat selection for resident and transient coyotes monitored from January 2015 to June 2017 in the tristate region of Alabama, Georgia, and South Carolina. Error bars shown represent standard error

We evaluated 1,501 monthly movement paths of individual coyotes (900 residents and 601 transients), with the number of individuals included in each month ranging from 52 to 74 individuals. We observed high FPT values (ARS behaviors) in all monthly movement datasets analyzed, and the average radius at which ARS behaviors occurred varied considerably across months (Figure 4). Modeling analyses revealed that all land cover variables affected ARS behaviors throughout the year; however, which variables were important and the direction of correlation (i.e., positive or negative) varied among months (Tables 1 and 2; Figure 5). Resident coyote FPT values were positively correlated with agriculture during fall and winter months, suggesting residents were more likely to engage in ARS behaviors near agricultural land cover during these months; however, FPT values were negatively correlated with agriculture during spring months. Resident FPT values were negatively correlated with developed habitats during May–August, deciduous land cover during June–August, and wetlands during September–January except during November (Figure 5). Edge density was positively correlated with resident FPT values in all months except April, June, July, and October (see Figure 5). Transient coyote FPT values were negatively correlated with developed areas throughout much of the year, suggesting transients were more likely to engage in ARS behavior near developed areas (Figure 5). Transient FPT values were also negatively correlated with wetlands during July–September. Transient FPT values were negatively correlated with agriculture across most months except June and July. Edge density was positively correlated with transient FPT values in all months except March, August, and November (Figure 5).

**TABLE 1:** Beta coefficient, standard error, *t* value, and *p* value estimates of the top‐ranked generalized linear mixed model (GLMM) estimating FPT values for resident coyotes monitored from January 2015 to June 2017 in Alabama, Georgia, and South Carolina

Month	habitat type	Beta estimate	*SE*	*t* Value	*p* Value
January	Deciduous	−63.69	23.82	−2.67	<.001
	Wetland	28.47	10.94	2.60	<.001
	Cropland	−19.35	7.79	−2.48	<.001
	Develop	60.12	10.73	5.60	<.001
	Pine Forest	98.00	10.73	4.19	<.001
	Edge Density	68.24	23.38	2.88	<.001
February	Deciduous	58.10	23.77	2.45	.014
	Wetland	−28.47	11.39	−2.49	.014
	Cropland	−20.92	7.25	−2.89	.003
	Develop	−7.99	10.31	−0.78	.04
	Pine Forest	−129.94	24.10	−5.39	<.001
	Edge Density	12.94	1.65	9.33	<.001
March	Deciduous	−63.15	17.87	−3.53	<.001
	Wetland	−19.05	8.32	−2.28	.02
	Cropland	−26.01	4.48	−5.80	<.001
	Develop	1.42	7.98	0.18	.88
	Pine Forest	26.19	18.54	1.40	.17
	Edge Density	14.26	6.25	2.34	.02
April	Deciduous	37.54	27.86	1.35	.1
	Wetland	−39.42	11.68	−3.38	<.001
	Cropland	75.06	4.72	15.89	<.001
	Develop	10.88	12.44	0.87	.34
	Pine Forest	−26.36	27.98	−0.94	.38
	Edge Density	24.25	18.77	1.01	.27
May	Deciduous	13.78	19.95	0.69	.49
	Wetland	−17.23	8.37	−2.06	.04
	Cropland	11.18	3.25	3.44	<.001
	Develop	32.69	9.41	3.47	<.001
	Pine Forest	−35.92	20.65	−1.74	.08
	Edge Density	19.16	2.12	1.24	<.001
June	Deciduous	72.92	21.56	3.38	<.001
	Wetland	15.33	9.30	1.65	.09
	Cropland	70.38	3.51	20.07	<.001
	Develop	31.91	10.28	3.10	.001
	Pine Forest	−1.16	23.77	−0.05	.9
	Edge Density	4.44	0.74	0.72	.06
July	Deciduous	42.52	21.69	1.96	.04
	Wetland	−56.07	9.30	−6.03	<.001
	Cropland	−2.39	3.41	−0.70	.48
	Develop	95.29	9.54	9.99	<.001
	Pine Forest	−26.90	23.06	−1.17	.24
	Edge Density	15.89	11.19	1.02	.35
August	Deciduous	38.17	26.92	1.42	.04
	Wetland	−17.59	10.87	−1.62	<.001
	Cropland	3.74	3.42	1.09	.48
	Develop	81.31	12.60	6.45	<.001
	Pine Forest	49.00	29.28	1.67	.24
	Edge Density	22.28	1.89	9.79	<.001
September	Deciduous	32.08	25.02	1.28	.19
	Wetland	50.70	10.23	4.95	<.001
	Cropland	12.49	3.61	3.46	<.001
	Develop	−17.29	13.74	−1.26	.2
	Pine Forest	−93.09	27.41	−3.40	<.001
	Edge Density	68.62	14.61	4.41	<.001
October	Deciduous	−6.52	26.18	−0.25	.80
	Wetland	30.49	11.35	2.69	.007
	Cropland	−12.89	4.04	−3.19	.001
	Develop	8.18	13.20	0.62	.54
	Pine Forest	−46.80	27.93	−1.68	.09
	Edge Density	−1.24	6.77	0.23	.66
November	Deciduous	60.39	21.78	2.77	.005
	Wetland	−3.27	9.29	−0.35	.73
	Cropland	−33.12	5.17	−6.40	<.001
	Develop	−45.65	10.34	−4.10	<.001
	Pine Forest	38.05	21.42	1.77	<.001
	Edge Density	12.28	2.38	8.21	<.001
December	Deciduous	3.08	30.18	0.10	.92
	Wetland	44.39	12.53	3.54	<.001
	Cropland	−49.03	8.03	−6.11	<.001
	Develop	26.29	13.68	1.92	.05
	Pine Forest	30.75	29.22	1.05	.29
	Edge Density	52.97	6.05	5.78	<.001

**TABLE 2:** Beta coefficient, standard error, *t* value, and *p* value estimates of the top‐ranked generalized linear mixed model (GLMM) estimating the relationship between FPT values and land cover type for transient coyotes monitored from January 2015 to June 2017 in Alabama, Georgia, and South Carolina

Month	Habitat type	Beta estimate	*SE*	*t* Value	*p* Value
January	Deciduous	−147.84	13.52	−10.94	<.001
	Wetland	31.99	10.90	2.94	.003
	Cropland	37.99	4.79	7.92	<.001
	Develop	10.29	11.12	0.93	.35
	Pine Forest	54.46	18.27	2.98	.002
	Edge Density	21.28	5.68	4.67	<.001
February	Deciduous	−78.59	12.97	−6.06	<.001
	Wetland	15.16	5.67	2.67	.007
	Cropland	42.24	2.25	18.79	<.001
	Develop	−20.49	6.80	−3.01	.002
	Pine Forest	22.53	10.36	2.18	.02
	Edge Density	33.84	3.71	15.45	<.001
March	Deciduous	−95.95	14.47	−6.63	<.001
	Wetland	19.06	6.18	3.08	.002
	Cropland	7.66	2.10	3.64	<.001
	Develop	−3.93	6.55	−0.59	.55
	Pine Forest	−8.49	10.50	0.81	.42
	Edge Density	5.58	2.72	1.72	.06
April	Deciduous	−31.82	11.13	−3.55	1.44
	Wetland	−6.96	5.73	−1.41	.16
	Cropland	10.33	2.14	6.31	<.001
	Develop	15.48	6.06	2.87	.004
	Pine Forest	96.24	12.36	10.44	<.001
	Edge Density	19.51	2.63	16.35	<.001
May	Deciduous	−5.53	8.39	−1.66	.62
	Wetland	1.54	6.26	−0.71	.79
	Cropland	7.96	2.07	−2.09	<.001
	Develop	24.11	6.62	−2.55	<.001
	Pine Forest	−8.70	13.38	−6.22	.48
	Edge Density	16.94	4.34	3.12	<.001
June	Deciduous	−13.94	8.39	−1.66	.09
	Wetland	−4.45	6.26	−1.71	.48
	Cropland	−4.32	2.07	−2.09	.04
	Develop	−16.85	6.62	−2.55	.01
	Pine Forest	−83.18	13.38	−6.22	<.001
	Edge Density	3.16	1.89	1.22	.04
July	Deciduous	13.84	6.25	2.22	.02
	Wetland	−25.03	6.09	−4.11	<.001
	Cropland	−5.38	2.14	−2.52	.012
	Develop	−55.88	6.58	−8.49	<.001
	Pine Forest	49.43	10.71	4.61	<.001
	Edge Density	24.37	8.81	2.69	<.001
August	Deciduous	−15.30	10.35	−1.48	.14
	Wetland	−28.19	6.02	−4.69	<.001
	Cropland	4.82	2.01	2.40	.01
	Develop	−21.81	7.04	−3.05	.002
	Pine Forest	−5.46	13.05	−0.42	.68
	Edge Density	−0.23	6.92	−0.02	.95
September	Deciduous	−20.80	16.49	−1.26	.21
	Wetland	−15.09	7.88	−1.91	.05
	Cropland	44.99	2.85	15.76	<.001
	Develop	−69.98	9.48	−7.38	<.001
	Pine Forest	75.71	15.30	4.95	<.001
	Edge Density	20.61	3.19	8.64	<.001
October	Deciduous	−13.05	8.52	−1.53	.13
	Wetland	2.18	4.56	0.48	.63
	Cropland	2.79	1.64	1.69	.09
	Develop	−18.86	6.15	−3.06	.002
	Pine Forest	15.71	10.13	1.56	.12
	Edge Density	9.48	1.83	6.34	<.001
November	Deciduous	5.27	12.83	4.11	<.001
	Wetland	−4.22	7.68	−0.05	.96
	Cropland	3.51	3.23	10.72	<.001
	Develop	−2.10	9.83	−2.13	.033
	Pine Forest	2.53	15.43	1.64	.11
	Edge Density	1.71	0.94	1.27	.34
December	Deciduous	148.06	21.68	6.83	<.001
	Wetland	−94.08	11.21	−8.39	<.001
	Cropland	7.46	4.77	1.56	.12
	Develop	−19.25	13.15	−1.46	.14
	Pine Forest	57.99	18.77	3.09	.002
	Edge Density	48.26	25.33	2.28	.002

## 4 DISCUSSION

We found that high FPT values (ARS behaviors) of coyotes correlated to specific land cover types across two large study areas in the Southeast, suggesting both resident and transient coyotes used particular habitats to engage in ARS behaviors such as foraging or loafing. Our results supported our first hypothesis that differences in territorial strategy (resident vs. transient) impacted space use and FPT values in relation to habitat characteristics. We found substantive variation in the direction and magnitude of correlations between high FPT values (ARS behaviors) and land cover type across months for both residents and transients, implying considerable temporal variation in individual behavior. This finding is not entirely surprising, as habitat selection by coyotes has been shown to be highly variable and context dependent, even for resident individuals (Gosselink et al., 2003; Harrison et al., 1991; Patterson & Messier, 2001). Additionally, contrary to traditional RSF approaches that rely solely on an animal's physical location to infer selection or use of particular habitats, FPT analysis accounts for the animal's movement path and associates physical locations with biological activities such as foraging or dinning (Fauchald & Tverra, 2003). Thus, although coyotes may be more likely to be near particular habitats throughout time, our findings suggest they are likely engaging in ARS behaviors in a diversity of habitats, reflecting their behavioral plasticity and generalist foraging strategy (Gosselink et al., 2003; Hinton et al., 2017; Ward et al., 2018).

### 4.1 Movement rates

We observed that movement rates varied across months for both residents and transients, although transient movement rates were greater than those of residents in all months except January. Previous work has found that transients typically have larger ranges (Hinton et al., 2015; Kamler & Gipson, 2000) and greater movement rates than residents (Sasmal et al., 2019). Our estimated monthly movement rates of coyotes were generally less than those previously reported in other studies for both residents (165.5–202.1 m/h vs. 295.3–449.8 m/h; Sasmal et al., 2019) and transients (183.4–229.7 m/h vs. 283.0–488.5 m/h; Sasmal et al., 2019). These differences likely arise from differences in classification criteria for residents and transients, as well as differences in the temporal scale at which movement rates were calculated between studies (differences in relocation fix rate and monthly vs. seasonal study periods) and our increased sample size, which would minimize the effect of outlier movement steps (i.e., long‐distance dispersal). Residents had lower movement rates during breeding and pup‐rearing season (March–August), with the lowest movement rates in June, a time when pups are likely emerging from the den yet still have limited mobility, thus indirectly limiting mobility of adults caring for pups (Andelt, 1985). Residents had the greatest movement rates during September, likely coinciding with dispersal of pups from their natal range (Andelt, 1985; Bekoff & Wells, 1986). Transients also had greatest movement rates during September, but exhibited relatively high movement rates throughout much of the year, with the lowest movement rates occurring in January (183.4 m/h; Figure 1b).

### 4.2 First passage time analysis

Previous research has found clear patterns of habitat selection in both resident and transient coyotes (Hinton et al., 2015; Holzman et al., 1992; Kamler & Gipson, 2000). Transient coyotes were previously found to be more likely to select for human‐disturbed habitats such as roads (Hinton et al., 2015) and urban development (Gerhrt et al., 2009). Similarly, we found that transient ARS behaviors were more likely to occur near developed areas during February and June–October, supporting our prediction that transient high FPT values (ARS behaviors) would be correlated with human developments. Conversely, resident FPT values were negatively correlated with developed areas during January and May–September, again supporting our predictions. Importantly, this time period overlaps with when individuals may be rearing pups (April–Sept; Bekoff & Wells, 1986; Kilgo et al., 2017), an activity only resident coyotes engage in (Gese, 2004; Mills & Knowlton, 1991). High FPT values associated with resident ARS behaviors during these months are likely a combination of denning/whelping (Mar–May), pup‐rearing (May–Sept), and foraging (year‐round) behaviors. Due to our large sample size and study extent, we did not attempt to empirically quantify whether resident animals successfully reproduced each year of monitoring, and thus we cannot differentiate between these behaviors. However, avoidance of developed areas by residents, and by proxy human activities known to increase mortality risk (Kitchen et al., 2000), during pup‐rearing may be a strategy to increase survival of both parents and pups (Figure 5).

We found resident high FPT values (ARS behaviors) were generally more likely to occur near wetlands from February–August (excluding June), which encompasses breeding (Jan–March) and pup‐rearing seasons (April–Sept) for coyotes. Residents with offspring are limited in their movements by the relatively reduced mobility of young pups (Andelt, 1985; Gese, 2004). Focusing foraging and pup‐rearing activities closer to wetlands and free water sources may decrease energetic costs associated with accessing water sources for both themselves and their offspring. Additionally, transient ARS behaviors were more likely to occur near wetlands from July to September. Resident and transient selection for wetlands overlaps with the warm summer months when the risk of heat stress for both is higher, and access to water for hydration and thermoregulation can mitigate this risk for both adults and (for residents) pups (Afik & Pinshow, 1993). Likewise, edge density was generally an important variable for both residents and transients, and the correlation between ARS behaviors and edge density was always positive when it is was significant. This finding supports previous work, indicating edge habitats provide important foraging opportunities for coyotes (Heske et al., 1999; Hinton et al., 2015; Ward et al., 2018).

Importantly, FPT analysis is known to be dependent on scale (Byrne & Chamberlain, 2012; Frair et al., 2005), with periods of ARS behavior potentially nested within larger periods of restricted movement behavior along an animal's movement path. The temporal scale of our movement data, where locations were collected every 4 h, allowed for extended monitoring of individuals by prolonging transmitter battery life but may have masked fine‐scale movement behaviors that could have influenced FPT estimation. Specifically, the 4‐hour fix rate interval has the potential to overestimate FPT by missing sinuous movements made during the interval between fixes. However, because the fix rate and FPT estimation methods (interpolation distance, range of scales at which FPT was estimated, etc.) was constant across all individuals, we are confident our methods allow for robust estimation of FPT at the temporal scale of our data and allow for accurate comparison among individuals. Additionally, the scale of ARS behaviors can be influenced by several different factors including habitat configuration and territoriality (Byrne & Chamberlain, 2012; Fauchald & Tverra, 2003; Frair et al., 2005). We quantified the spatial relationship between individual ARS behaviors and land cover types using a distance‐based approach to maintain consistency across individuals; however, this approach has limited ability to assess true habitat configuration relative to proportion‐based approaches (i.e., quantifying the proportion of each land cover type within a set area, such as the FPT radius used in each monthly analysis). However, the distance‐based approach allowed for consistent quantification among resident and transient individuals across months, making it the most appropriate approach for our study. Additionally, territoriality can influence the ability to delineate ARS behaviors because an individual may restrict its movements to within its home range due to territorial boundaries, and not necessarily because of ARS behaviors (e.g., foraging). All resident coyotes in our study could easily traverse their estimated home range during the month period at which we estimated FPT, allowing for the possibility that an animal may turn back on its path as it moves among portions of its home range in addition to foraging or resting behaviors. However, our research objectives were not to infer specific behaviors (i.e., foraging vs. denning vs. resting) within periods of ARS behavior, but rather to associate general ARS behavior associated with high FPT values with land cover characteristics for both resident and transient animals. Furthermore, the substantive variation in space use and movement rates among resident and transient animals and the temporal scale of our movement data (i.e., relocations every 4 h) likely indicate that patterns associated with inferred behavioral states may also vary widely among individuals. Regardless, we believe that the resolution of our analysis and spatial scale at which we inferred ARS behaviors were sufficient and appropriate to elucidate land cover characteristics associated with these behaviors.

The complex, variable patterns in space use and movement behaviors of both residents and transients make effective, continued management of coyotes difficult, especially at a landscape scale. Although we found clear evidence of spatiotemporal patterns associated with ARS behaviors for both resident and transient animals, the substantive variation among individual coyotes indicates that broad, generalized management actions (e.g., removal) may not be appropriate for targeting coyotes at a population level. Indeed, previous research has found that large‐scale management efforts in the Southeast are rarely successful at long‐term management of coyote populations (Kilgo et al., 2014; Kirepka et al., 2017). Rather, management actions are likely to be more effective at small scales when individual patterns of movement behavior are known.

## CONFLICT OF INTEREST

The authors have no competing interests to declare.

## AUTHOR CONTRIBUTIONS

**Sarah C. Webster:** Conceptualization (equal); Formal analysis (lead); Investigation (equal); Methodology (lead); Software (lead); Writing – original draft (lead); Writing – review & editing (equal). **James C. Beasley:** Writing – review & editing (equal). **Joseph W. Hinton:** Investigation (equal); Writing – review & editing (equal). **Michael J. Chamberlain:** Conceptualization (equal); Funding acquisition (lead); Writing – review & editing (equal).

## Data Availability

Animal movement data can be found online at MoveBank (MoveBank ID: 1966782762).

## APPENDIX 1

Akaike Information Criterion (AIC) model rankings for generalized linear mixed models (GLMMs) estimating the relationship between FPT values and landcover type for transient coyotes monitored from January 2015 – June 2017 in Alabama, Georgia, and South Carolina. K = number of parameters, AIC = Akaike Information Criterion, ΔAIC = Delta AIC, AICWt = AIC weight, LL = negative log likelihood, and Adj‐*R* ^2^ = adjusted *R* ^2^ value (provided only for top ranked models)

**Table:**

Status	Month	Model	*K*	AIC	ΔAIC	AICWt	LL	Adj‐*R* ^2^
Transient	January	Full Model^a^	8	108978.1	0	1	−54481.05	.44
		Decid^b^ + Pine^c^ + Wet^d^	6	109050	71.9	0	−54519.01	–
		Decid + Pine	5	109063.3	85.16	0	−54526.65	–
		Crop^e^ + Edge^f^ + Dev^g^	6	109111.2	133.03	0	−54549.58	–
		Edge + Dev	5	109117.8	139.69	0	−54553.91	–
		Crop + Edge	5	109175.1	196.95	0	−54582.54	–
	February	Full Model	8	248559	0	1	−124271.5	.56
		Crop + Edge + Dev	6	248608	49.05	0	−124298	–
		Edge + Dev	5	248615.2	56.24	0	−124302.6	–
		Decid + Pine + Wet	6	248916.8	357.87	0	−124452.4	–
		Decid + Pine	5	248933.5	374.55	0	−124461.8	–
		Crop + Edge	5	248976.2	417.28	0	−124483.1	–
	March	Full Model	8	287154.4	0	1	−143569.2	.47
		Decid + Pine + Wet	6	287173	18.57	0	−143580.5	–
		Decid + Pine	5	287186.2	31.76	0	−143588.1	–
		Crop + Edge + Dev	6	287208.1	53.7	0	−143598.1	–
		Edge + Dev	5	287216.6	62.15	0	−143603.3	–
		Crop + Edge	5	287226	71.63	0	−143608	–
	April	Full Model	8	256393	0	1	−128188.5	.23
		Decid + Pine + Wet	6	256446.4	53.47	0	−128217.2	–
		Decid + Pine	5	256452.9	59.97	0	−128221.5	–
		Crop + Edge	5	256458.5	65.5	0	−128224.2	–
		Crop + Edge + Dev	6	256514.7	121.7	0	−128251.3	–
	May	Full Model	8	208779.6	0	0.99	−104381.8	.66
		Crop + Edge + Dev	6	208790	10.35	0.01	−104389	–
		Edge + Dev	5	208793.3	13.72	0	−104391.7	–
		Crop + Edge	5	208803	23.38	0	−104396.5	–
		Decid + Pine + Wet	6	208815.5	35.88	0	−104401.7	–
		Decid + Pine	5	208819.1	39.47	0	−104404.5	–
	June	Full Model	8	180901.4	0	1	−90442.71	.38
		Decid + Pine + Wet	6	180918.1	16.67	0	−90453.05	–
		Crop + Edge	5	180918.6	17.14	0	−90454.29	–
		Decid + Pine	5	180921.6	20.21	0	−90455.82	–
		Crop + Edge + Dev	6	180953	51.52	0	−90470.47	–
		Edge + Dev	5	180957.2	55.75	0	−90473.59	–
	July	Full Model	8	161494.9	0	1	−80739.45	.35
		Crop + Edge + Dev	6	161529	34.11	0	−80758.51	–
		Crop + Edge	5	161529.5	34.52	0	−80759.72	–
		Edge + Dev	5	161546.3	51.39	0	−80768.16	–
		Decid + Pine + Wet	6	161582.5	87.63	0	−80785.27	–
		Decid + Pine	5	161595	100.04	0	−80792.48	–
	August	Full Model	8	159770.3	0	1	−79877.13	.27
		Crop + Edge + Dev	6	159782.3	12.03	0	−79885.15	–
		Decid + Pine + Wet	6	159789.3	19.03	0	−79888.65	–
		Edge + Dev	5	159809.9	39.61	0	−79899.94	–
		Decid + Pine	5	159810.1	39.79	0	−79900.03	–
		Crop + Edge	5	159811.8	41.49	0	−79900.88	–
	September	Full Model	8	137063.2	0	1	−68523.57	.30
		Crop + Edge + Dev	6	137098.5	35.31	0	−68543.23	–
		Edge + Dev	5	137107.3	44.16	0	−68548.66	–
		Crop + Edge	5	137323.2	260.05	0	−68656.6	–
		Decid + Pine + Wet	6	137348.2	285.02	0	−68668.08	–
		Decid + Pine	5	137352.2	289.08	0	−68671.12	–
	October	Full Model	8	133713.8	0	1	−66848.86	.11
		Crop + Edge + Dev	6	133726.4	12.64	0	−66857.18	–
		Crop + Edge	5	133727.1	13.37	0	−66858.55	–
		Edge + Dev	5	133729.3	15.54	0	−66859.64	–
		Decid + Pine + Wet	6	133730.2	16.42	0	−66859.08	–
		Decid + Pine	5	133734.2	20.44	0	−66862.09	–
	November	Full Model	8	122958.7	0	1	−61471.35	.24
		Crop + Edge + Dev	6	122992.8	34.05	0	−61490.38	–
		Edge + Dev	5	122996.8	38.03	0	−61493.37	–
		Decid + Pine + Wet	6	123080.9	122.2	0	−61534.45	–
		Decid + Pine	5	123085.5	126.75	0	−61537.73	–
		Crop + Edge	5	123100.4	141.71	0	−61545.21	–
	December	Full Model	8	100444	0	1	−50213.97	.48
		Decid + Pine + Wet	6	100456.2	12.2	0	−50222.08	–
		Crop + Edge + Dev	6	100509.8	65.82	0	−50248.89	–
		Decid + Pine	5	100528.1	84.07	0	−50259.02	–
		Crop + Edge	5	100561.2	117.22	0	−50275.59	–
		Edge + Dev	5	100574	130	0	−50281.98	–
Resident	January	Full Model	8	172304	0	1	−86143.98	.53
		Crop + Edge + Dev	6	172322.1	18.14	0	−86155.05	–
		Edge + Dev	5	172329.3	25.35	0	−86159.65	–
		Decid + Pine + Wet	6	172335.9	31.87	0	−86161.92	–
		Decid + Pine	5	172342.4	38.42	0	−86166.19	–
		Crop + Edge	5	172348.1	44.17	0	−86169.07	–
	February	Full Model	8	366361	0	1	−183172.5	.52
		Crop + Edge + Dev	6	366376.8	15.78	0	−183182.4	–
		Edge + Dev	5	366381.6	20.59	0	−183185.8	–
		Decid + Pine + Wet	6	366392.2	31.19	0	−183190.1	–
		Decid + Pine	5	366396.5	35.48	0	−183193.2	–
		Crop + Edge	5	366401.7	40.71	0	−183195.9	–
	March	Full Model	8	412732.1	0	1	−206358	.35
		Crop + Edge + Dev	6	412745.5	13.41	0	−206366.8	–
		Edge + Dev	5	412752.6	20.51	0	−206371.3	–
		Decid + Pine + Wet	6	412771.4	39.32	0	−206379.7	–
		Crop + Edge	5	412772.3	40.18	0	−206381.1	–
		Decid + Pine	5	412779.5	47.41	0	−206384.8	–
	April	Full Model	8	362301.7	0	1	−181142.9	.38
		Crop + Edge + Dev	6	362315.1	13.42	0	−181151.6	–
		Edge + Dev	5	362323.1	21.39	0	−181156.5	–
		Decid + Pine + Wet	6	362332.9	31.17	0	−181160.4	–
		Crop + Edge	5	362337.2	35.48	0	−181163.6	–
		Decid + Pine	5	362341.6	39.85	0	−181165.8	–
	May	Full Model	8	311526	0	1	−155755	.39
		Crop + Edge + Dev	6	311538.9	12.91	0	−155763.5	–
		Edge + Dev	5	311544.1	18.1	0	−155767	–
		Decid + Pine + Wet	6	311559.4	33.41	0	−155773.7	–
		Crop + Edge	5	311564.4	38.42	0	−155777.2	–
		Decid + Pine	5	311564.7	38.7	0	−155777.3	–
	June	Full Model	8	255054.8	0	1	−127519.4	.45
		Crop + Edge + Dev	6	255071.9	17.03	0	−127529.9	–
		Edge + Dev	5	255076.7	21.9	0	−127533.4	–
		Decid + Pine + Wet	6	255089.3	34.5	0	−127538.7	–
		Decid + Pine	5	255094.6	39.81	0	−127542.3	–
		Crop + Edge	5	255095.9	41.07	0	−127542.9	–
	July	Full Model	8	228091.6	0	1	−114037.8	.53
		Crop + Edge + Dev	6	228104.9	13.29	0	−114046.4	–
		Edge + Dev	5	228111.2	19.57	0	−114050.6	–
		Decid + Pine + Wet	6	228125.6	34.03	0	−114056.8	–
		Decid + Pine	5	228131.9	40.28	0	−114060.9	–
		Crop + Edge	5	228133.1	41.46	0	−114061.5	–
	August	Full Model	8	224832.4	0	1	−112408.2	.31
		Crop + Edge + Dev	6	224846	13.58	0	−112417	–
		Edge + Dev	5	224852.9	20.45	0	−112421.4	–
		Decid + Pine + Wet	6	224858	25.53	0	−112423	–
		Crop + Edge	5	224864	31.61	0	−112427	–
		Decid + Pine	5	224865	32.57	0	−112427.5	–
	September	Full Model	8	183486.7	0	1	−91735.34	.37
		Crop + Edge + Dev	6	183501.8	15.13	0	−91744.9	–
		Decid + Pine + Wet	6	183505	18.25	0	−91746.47	–
		Edge + Dev	5	183507.1	20.44	0	−91748.56	–
		Decid + Pine	5	183510.2	23.55	0	−91750.12	–
		Crop + Edge	5	183511.4	24.74	0	−91750.71	–
	October	Full Model	8	188601.5	0	1	−94292.75	.45
		Crop + Edge + Dev	6	188616.9	15.36	0	−94302.43	–
		Edge + Dev	5	188622.5	20.97	0	−94306.24	–
		Crop + Edge	5	188623.8	22.31	0	−94306.91	–
		Decid + Pine + Wet	6	188626.9	25.4	0	−94307.45	–
		Decid + Pine	5	188632.1	30.61	0	−94311.06	–
	November	Full Model	8	179386.9	0	1	−89685.45	.32
		Crop + Edge + Dev	6	179400.9	14.02	0	−89694.46	–
		Edge + Dev	5	179406	19.08	0	−89697.99	–
		Decid + Pine + Wet	6	179416.7	29.81	0	−89702.36	–
		Crop + Edge	5	179421.8	34.87	0	−89705.89	–
		Decid + Pine	5	188632.1	9245.21	0	−94311.06	–
	December	Full Model	8	143644.5	0	1	−71814.25	.38
		Crop + Edge + Dev	6	143658.8	14.29	0	−71823.4	–
		Decid + Pine + Wet	6	143663.7	19.21	0	−71825.86	–
		Edge + Dev	5	143664	19.5	0	−71827.01	–
		Crop + Edge	5	143667.5	23.02	0	−71828.77	–
		Decid + Pine	5	143669	24.49	0	−71829.5	–

^a^ Global model with all variables.

^b^ Mixed Deciduous Forest.

^c^ Pine Forest.

^d^ Wetland.

^e^ Cropland.

^f^ Edge Density.

^g^ Developed.

## APPENDIX 2

Averaged estimates of monthly 95% ranges and 50% core areas for 171 resident and monthly 95% biding areas and 50% core biding areas for transient coyotes monitored from January 2015 – Jun 2017 in the tri‐state region of Alabama, Georgia, and South Carolina, USA

**Table:**

Territorial Status	Month	95% Ranges (km^2^)	SD	50% Core Areas (km^2^)	*SD*
Resident	January	21.28	36.07	4.51	7.54
	February	30.51	11.70	4.99	15.20
	March	13.55	11.07	2.67	2.34
	April	14.98	21.68	2.67	2.67
	May	11.19	9.03	1.99	2.12
	June	13.55	17.24	2.64	4.01
	July	12.02	8.96	2.17	1.81
	August	11.97	9.41	2.51	1.92
	September	12.08	6.48	2.83	1.78
	October	12.59	8.87	2.97	2.31
	November	13.56	9.97	3.04	1.90
	December	14.66	12.03	3.42	2.68
Transient	January	202.14	319.23	41.22	68.05
	February	561.44	1209.62	128.21	296.28
	March	480.16	994.50	121.71	283.03
	April	355.87	827.62	83.52	226.82
	May	321.16	498.47	72.08	112.36
	June	377.04	1033.13	95.77	278.46
	July	349.52	678.56	82.90	219.06
	August	297.17	904.56	67.26	188.36
	September	449.67	1412.66	116.95	306.68
	October	387.23	924.21	95.88	279.92
	November	367.44	412.39	86.33	106.23
	December	276.90	382.69	57.25	89.60

## APPENDIX 3

Sample estimates for generalized linear mixed models comparing monthly movement rates between resident and transient coyotes monitored from January 2015 – June 2017 in the tri‐state region of Alabama, Georgia, and South Carolina, USA

**Table:**

Month	Resident mean movement rate (m/h)	Resident standard error	Transient mean movement rate (m/h)	Transient standard error	*t* Statistic	Degrees of freedom	*p* Value
Jan	183.01	2.47	183.43	4.25	0.61	13395	.54
Feb	173.62	2.14	211.46	3.38	10.25	19748	<.001
Mar	172.01	1.87	212.86	3.04	8.62	23708	<.001
Apr	169.35	1.78	223.27	3.14	6.23	23089	<.001
May	174.05	1.70	222.21	3.89	7.71	21954	<.001
Jun	165.52	1.68	223.21	4.41	9.25	19347	<.001
Jul	185.96	1.86	224.43	4.80	11.28	18570	<.001
Aug	189.35	1.99	208.92	3.62	7.38	17207	<.001
Sep	202.15	2.16	229.68	4.34	7.10	14662	<.001
Oct	194.10	2.18	215.66	4.03	3.68	14910	<.001
Nov	188.37	2.07	199.23	3.94	3.70	16089	<.001
Dec	187.21	2.32	193.26	4.84	13.86	13583	<.001
